# P-1105. Effectiveness of Imipenem-Relebactam for Multidrug-resistant *Pseudomonas aeruginosa* in Pneumonia and Bloodstream Infections in the United States (MIRAGE)

**DOI:** 10.1093/ofid/ofae631.1293

**Published:** 2025-01-29

**Authors:** Walaiporn Wangchinda, jason M Pogue, Lilian M Abbo, Megan Klatt, Ellen G Kline, Ryan C Kubat, Mayan Gilboa, Ana Vega, Yanbing Zhou, Emre Yucel, Ryan K Shields

**Affiliations:** University of Michigan College of Pharmacy, Ann Arbor, Michigan; University of Michigan, College of Pharmacy, Ann Arbor, MI; University of Miami Miller School of Medicine, Jackson Health System, Aventura, FL; The University of Kansas Health System, KS; University of Pittsburgh, Pittsburgh, Pennsylvania; University of Kansas, Kansas City, Kansas; University of Miami, Miami, Florida; Jackson Memorial Hospital, Miami, Florida; Merck, Rahway, New Jersey; Merck & Co., Inc., North Wales, Pennsylvania; University of Pittsburgh, Pittsburgh, Pennsylvania

## Abstract

**Background:**

Imipenem/relebactam (I/R) demonstrates potent *in vitro* activity against multidrug-resistant (MDR) *Pseudomonas aeruginosa*. The objective of this study is to evaluate the real-world effectiveness of I/R for treatment of MDR Pseudomonas infections across the U.S.

**Figure:**
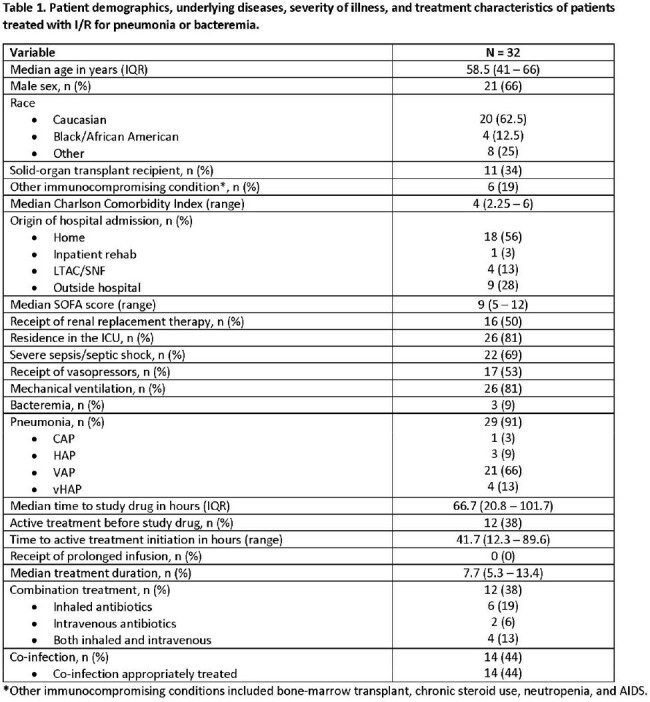

**Methods:**

MIRAGE is a retrospective, multicenter, observational study describing the real-world effectiveness of I/R against MDR *P. aeruginosa* pneumonia and bloodstream infections. Patients were included if they received I/R for >48h initiated within 7 days of the index MDR *P. aeruginosa* culture. Clinical success was defined at day 7 and 30 as survival, resolution of signs and symptoms of infection, and the absence of a recurrent infection due to MDR *P. aeruginosa*. At day 30, patients had to have also completed the intended treatment course. Patients who died within 48 hours, and those with cystic fibrosis or COVID-19 were excluded.

**Figure:**
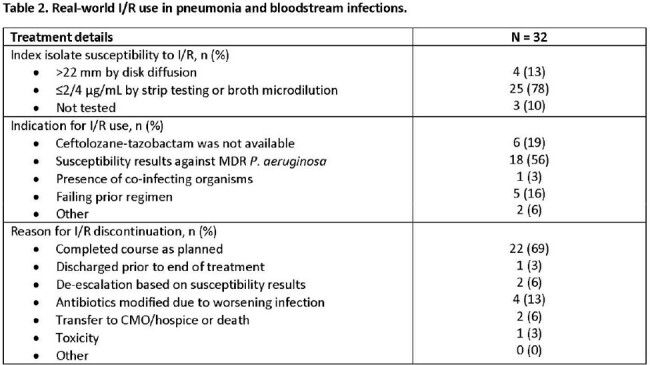

**Results:**

In a preliminary analysis, 32 patients from 4 centers met inclusion criteria (**Table 1).** The overall cohort was critically-ill; 53% and 81% were on vasopressors and mechanical ventilation at the time of I/R initiation, respectively. The median (IQR) SOFA score was 9 (5 – 12). 38% received treatment with a novel β-lactam for MDR P. aeruginosa infections prior to I/R. The median time to I/R initiation was 66.7 hours. Combination therapy with another *in vitro* active agent was used in 38%. The primary reason for I/R treatment was reported susceptibility results, which included resistance to other novel β-lactam agents (**Table 2**). 69% of patients completed the intended I/R treatment course. At day 7 and 30, 72% and 53% met criteria for clinical success, respectively (**Table 3**). The overall 30- and 90-day mortality rates were 22% and 31%, respectively. Recurrent infections were documented in 38% of patients within 90-days.

**Figure:**
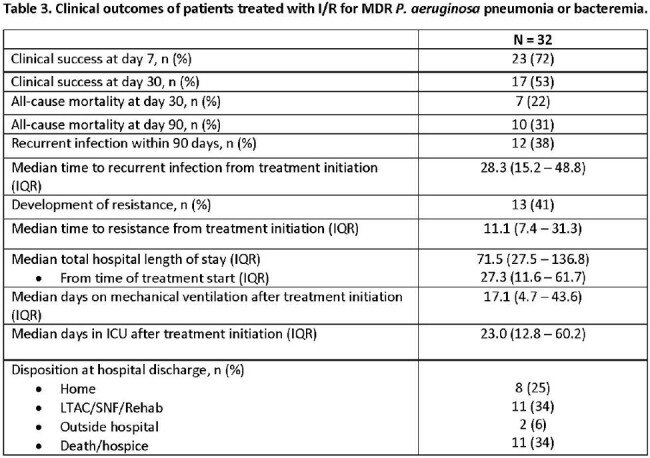

**Conclusion:**

In this critically-ill patient population we found that I/R was often used following treatment with other novel β-lactams. Clinical outcomes were generally comparable to those previously reported in real-world studies for alternative β-lactam/β-lactamase inhibitor agents. Thus, the data demonstrate that I/R plays an important role in treatment of MDR *P. aeruginosa* infections, particularly when other gents are not available or test resistant.

**Disclosures:**

**jason M. Pogue, PharmD**, Entasis: Advisor/Consultant|GSK: Advisor/Consultant|Melinta: Advisor/Consultant|Melinta: Grant/Research Support|Merck: Advisor/Consultant|Merck: Grant/Research Support|Shionogi: Advisor/Consultant|Shionogi: Grant/Research Support|Venatorx: Advisor/Consultant **Yanbing Zhou, PhD**, Merck: I am a full time Merck Employee and own stocks in the retirement plan provided by Merck.|Merck: Stocks/Bonds (Public Company) **Emre Yucel, PhD**, Merck: I am a full time Merck Employee and own stocks in the retirement plan provided by Merck.|Merck: Stocks/Bonds (Public Company) **Ryan K. Shields, PharmD, MS**, Allergan: Advisor/Consultant|Cidara: Advisor/Consultant|Entasis: Advisor/Consultant|GSK: Advisor/Consultant|Melinta: Advisor/Consultant|Melinta: Grant/Research Support|Menarini: Advisor/Consultant|Merck: Advisor/Consultant|Merck: Grant/Research Support|Pfizer: Advisor/Consultant|Roche: Grant/Research Support|Shionogi: Advisor/Consultant|Shionogi: Grant/Research Support|Utility: Advisor/Consultant|Venatorx: Advisor/Consultant|Venatorx: Grant/Research Support

